# *In vitro* study on role of σB protein in avian reovirus pathogenesis

**DOI:** 10.18632/oncotarget.24668

**Published:** 2018-04-13

**Authors:** Manas R. Praharaj, Aditya P. Sahoo, Tapan K.S. Chauhan, Ravi Kumar Gandham, Shikha Saxena, Ravi K. Agarwal, Kuldeep Dhama, Bina Mishra, Asok K. Marriappan, Ashok K. Tiwari, Puroshottam Prasad Goswami, Bishnu Prasad Mishra, Deepak Kumar

**Affiliations:** ^1^ Division of Veterinary Biotechnology, Indian Veterinary Research Institute, Izatnagar, Uttar Pradesh, India; ^2^ Division of Livestock Product Technology, Indian Veterinary Research Institute, Izatnagar, Uttar Pradesh, India; ^3^ Avian Disease Section, Division of Pathology, Indian Veterinary Research Institute, Izatnagar, Uttar Pradesh, India; ^4^ ICAR- Directorate on Foot and Mouth Disease, Mukteshwar, Nainital, Uttarakhand, India

**Keywords:** avian reovirus, S3 gene, σB protein, microarray, arthritis

## Abstract

Avian reoviruses, members of *Orthoreovirus* genus was known to cause diseases like tenosynovitis, runting-stunting syndrome in chickens. Among eight structural proteins, the proteins of S-class are mainly associated with viral arthritis but the significance of σB protein in arthritis is not established till date. In this infection pathological condition together with infection of joints often leads to arthritis because joints consists of cartilage which forms lubricating surface between two bones, and has limited metabolic, replicative and repair capacity.

To establish the role of σB protein in arthritis, an *in-vitro* microarray study was conducted consisting four groups viz. virus infected and control; pDsRed-Express-N1-σB and empty pDs-Red transfected, CEF cells. With cut-off value as FC ≥2, p value <0.05, 6709 and 4026 numbers of DEGs in virus and σB, respectively were identified. The Ingenuity Pathway Analysis gave an idea about the involvement of σB protein in “osteoarthritis pathway”, which was activated with z-score with 3.151. The pathway “Role of IL-17A in arthritis pathway” was also enriched with –log (p-value) 1.64. Among total 122 genes involved in osteoarthritis pathway, 28 upregulated and 11 downregulated DEGs were common to both virus and σB treated cells. Moreover, 14 upregulated and 7 downregulated were unique in σB transfected cells. Using qRT-PCR for IL-1B, BMP2, SMAD1, SPP1 genes, the microarray data was validated. We concluded that during ARV infection σB protein, if not fully partially leads to molecular alteration of various genes of host orchestrating the different molecular pattern in joints, leading to tenosynovitis syndrome.

## INTRODUCTION

ARV belongs to the family *Reoviridae*, genus *Orthoreovirus*. *Reoviridae*, a family of viruses which can affect the gastrointestinal tract as well as respiratory tract was first isolated from the respiratory and enteric tract of humans and animals but was not associated with the disease the name reovirus was used. The name is actually mnemonic for respiratory (r) enteric (e) orphan (o) virus. Genotype 1 reoviruses in poultry cause a severe joint condition popularly known as stunting and runting syndrome. Cartilage occupies an intermediate developmental state, as most bone develops from cartilage, but some cartilage is specifically arrested in this process (ossification) to provide the stable adult tissue. Chondrocytes are the only cell type within cartilage, secreting and enveloping themselves in a complex extracellular matrix mainly composed of type II collagen, aggrecan, hyaluronan, lubricin, and fibronectin.

The genome and protein compositions of ARV are generally similar to those of mammalian reovirus (MRV), the prototype of the *orthoreovirus* genus [1, 2]. However, some biological properties of the ARVs differ from mammalian reovirus, e.g. the lack of haemagglutination activity [3], the ability to induce fusion of cultured cells [4] and their pathogenicity towards their natural hosts. The avian reovirus genome expresses at least 12 primary translation products, of which 8 are structural proteins (σA, σB, σC, λA, λB, λC, μA, and μB) that become incorporated into progeny reovirions, other two, μBN and μBC, originate by post-translational cleavage of their precursor σB [5] and the other four proteins (μNS, σNS, p17 and p10) are non-structural, as they are not found in mature reovirions, but expressed in infected cells [6, 7]. σB protein produces group specific antibody against virus which plays an important role in viral pathogenesis [8].

Several methods for diagnosis of ARVs are reported [9, 10]. Additionally, virus isolation [11], immunofluorescent staining [12] and immunoperoxidase histochemistry [13, 14] offer the straight detection of viral antigens in tendon tissues. The methods for detection of ARV RNA from the cell culture [15] or from both cell cultures and tendon specimens [16] have been developed to provide a sensitive tool for the laboratory diagnosis. The serological diagnosis methods such as ELISA were also used for detection of serum antibodies in large number of samples simultaneously [17–20]. Molecular level diagnosis techniques include routine PCR [21, 22], multiplex PCR [23], reverse transcription loop-mediated isothermal amplification assay (RT-LAMP) [24], real time probe based loop mediated isothermal amplification (RT-Cy5 qLAMP) [25] etc.

The previous studies reported about the tenosynovitis/arthritis [26–28] and osteoporosis [29] induced by avian reovirus in younger stage of broiler poultry. Lameness in turkeys has sometimes been reported to be associated with avian reoviruses, but experimental evidence from the USA indicates the presence of novel reoviruses causing arthritis and tendon rupture clinically identical to that in chickens [30]. This disease causes acute lameness of bird affecting tibiotarsal-tarsometatarsal joint (hock joint), the main load-bearing joint of bird. The affected joints are swollen with rupture of gastrocnemius muscle in severe case accompanied with haemorrhage causing green colouration of skin at the joint. The main reason of mortality in birds is due to reduced growth and feed conversion.

Previously, the gene expression profiles of vero cells upon ARV S1133 infection and ARV-encoded pro-apoptotic protein σC over-expression was examined using microarray [31]. Further, a number of high throughput sequencing studies came in recent past. Identification and complete genome sequencing of two naturally occurring ARV variant strain co-infections having same M2 segment but were distantly evolved nine other segments in layer chickens using NGS was achieved [32]. Most recently, a gene expression RNA-Seq study showed that inoculation of chicken fibroblast DF-1 cells lines with ARV stimulates a prolonged antiviral response in host cells and interferes with cell growth and cell death pathways [33]. Despite many studies towards its diagnosis and pathogenesis induced by ARV, no study signifies the role of various genes of arthritis pathway expression in ARV and σB protein induced pathogenesis, which was the main objective of our study. For this, microarray along with IPA analysis was used with predesigned microarray chip to signify the probable mechanism of ARV and σB protein induced arthritic molecular changes. In this study, we analyzed changes in the expression of cellular genes in chicken embryo fibroblasts (CEFs) infected with the ARV DVB02 strain and σB transfection using microarray analysis. Analysis and functional studies of these genes and the relevant pathways may provide novel information that will increase our understanding of the pathogenesis of ARV and the mechanisms of *in-vitro* host responses.

## RESULTS

### Cloning confirmation and time course expression of pDsRed-Express-N1- σB in CEF cell

**Figure 1:**
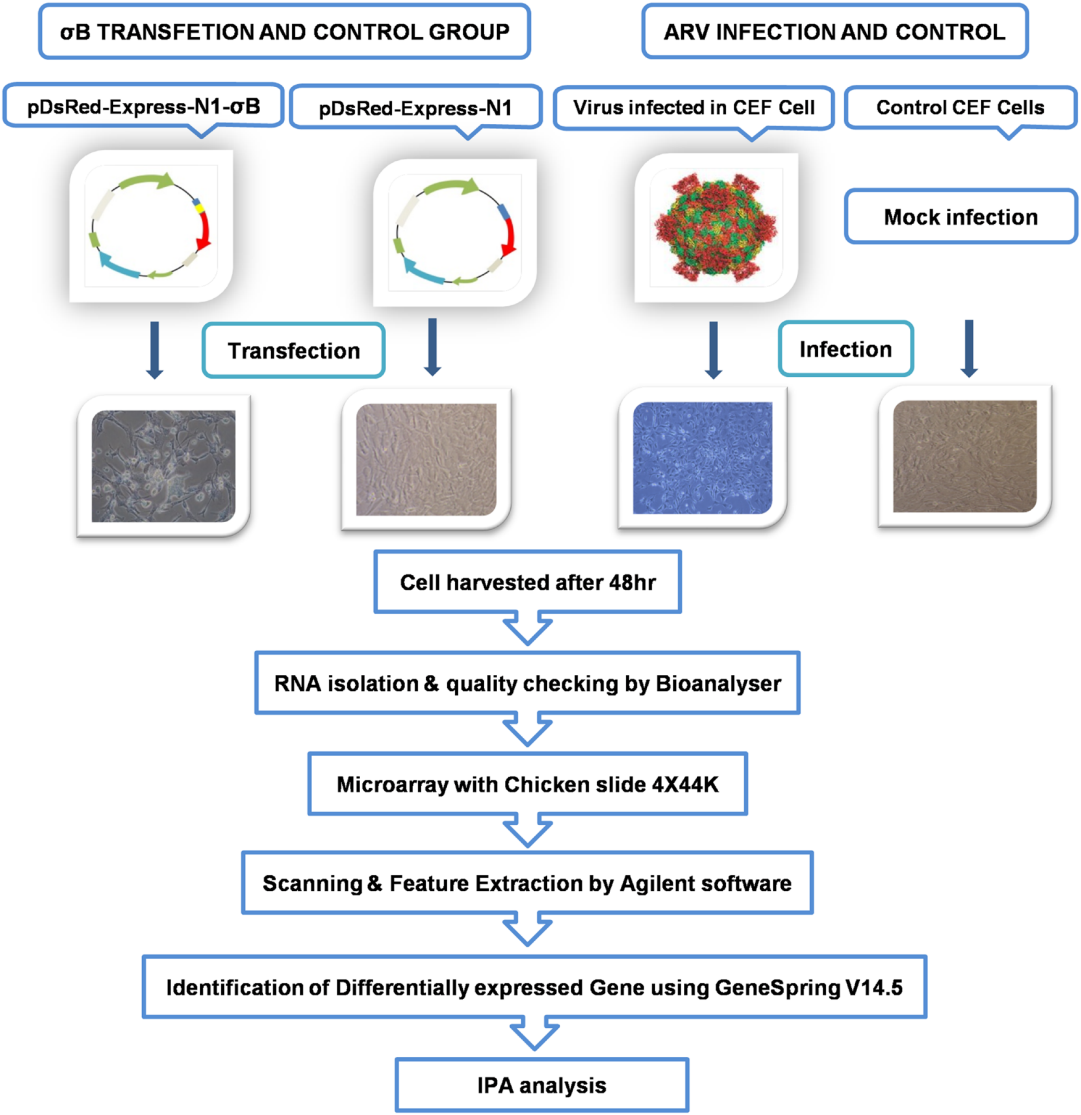
Schematic representation of workflow of microarray experiments for delineating osteoarthritic pathways in ARV and σB treated CEF cells

**Figure 2:**
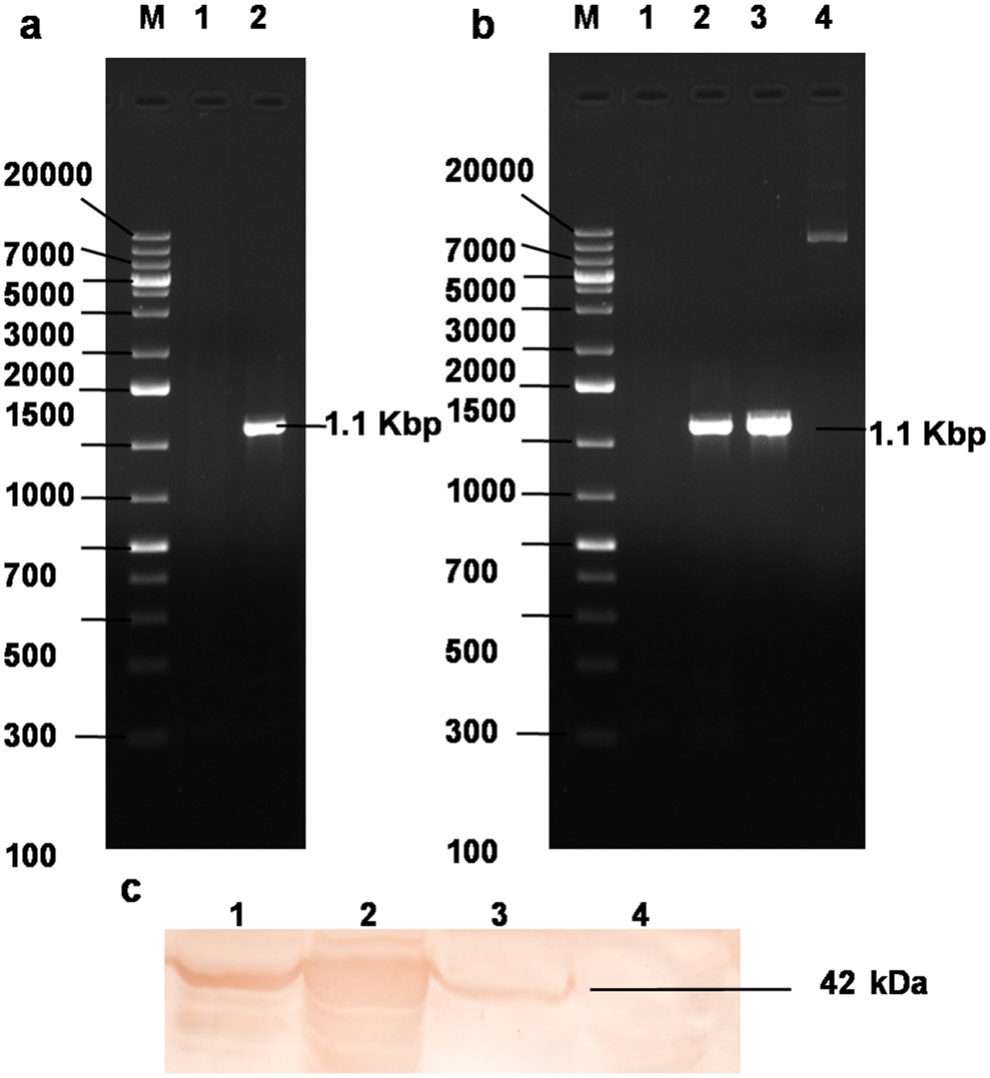
Cloning and expression of recombinant σB protein **(a)** PCR amplification of ARV σB gene. Lane M: 1 Kb plus DNA ladder, Lane 1: NTC, Lane 2: σB 1104 bp PCR product; **(b)** Lane M: 1 Kb plus DNA ladder, Lane 1: NTC, Lane 2-3: colony PCR yielding σB 1104bp PCR product; Lane 4: Plasmid construct pDsRed-Express-N1-σB; **(c)** Western blot analysis using hyperimmune sera rose against recombinant σB protein in SPF chicken. Lane 1: 24 h, Lane 2: *E. coli* expressed σB protein, Lane 3: 48 h, Lane 4: 72 h post pDsRed-Express-N1-σB plasmid transfection CEF cell lystae showing expressed eukaryotic σB protein.

The schematic overview of the microarray experiment is shown (Figure 1). The CEF adapted ARV field isolate (DVB02) available in the laboratory was revived and propagated under sterile conditions. The RNA was isolated; cDNA was synthesized. The σB gene was amplified at 1.1 kbp (Figure 2a) using S3-based PCR amplification by primers shown in Table 1. *Hind*III and *Bam*HI double digested pDsRed-Express-N1 expression vector and PCR amplified σB gene were ligated, transformed in *E. coli* cells and plated on LB agar plate. The colonies were selected and the recombinants were screened by colony PCR and further confirmed by double digestion by *Hind*III and *Bam*HI which released the desired fragment of 1.1 kbp (Figure 2b). The selected recombinant clone was abbreviated as pDsRed-Express-N1-σB. The plasmid DNA from the clone was transfected in 70-80% confluent cells in a four well plate using Lipofectamine 2000 (Invitrogen, USA) and cells were kept at 37°C in 5% CO_2_ concentration. Samples were collected at 24, 48, 72 h post transfection of pDsRed-Express-N1-σB and infection of ARV. After harvesting the cells from tissue culture plate, lysis of cells was done using mammalian protein extraction reagent (Genetix, India) according to manufacturer's protocol. Further, SDS-PAGE and western blot analysis showed the expression of 42 kDa σB protein using SPF chicken raised hyperimmune serum (Figure 2c). Optimum σB protein expression and virus titre was observed at 48 h post transfection which was further confirmed at transcript level by Real Time PCR analysis of σB transcripts. Thus, 48 h post transfection was chosen as suitable time point for microarray experiment.

**Table 1:** List of the genes and their forward & reverse primers

Gene	Primer sequence	Accession number	Product length (bp)
SPP1	F 5’-CGAAGATCGCCACAGCATTG-3’	NM_204535	137 (860-996)
	R 5’-CAAACACACGTCGCTATGGC-3’		
BMP2	F 5’-ATGACGTGGGGTGGAATGAC-3’	NM_204358	164 (905-1068)
	R 5’-GCAAGCCTTGGGGATTTTGG-3’		
SMAD1	F 5’-TGGAATGCTGCGAGTTTCCT-3’	NM_001201455	138 (317-454)
	R 5’-GGCTGTGCTGAGGGTTGTAT-3’		
IL1B	F 5’-CGCTTCATCTTCTACCGCCT-3’	NM_204524	144 (666-809)
	R 5’-GATGTTGACCTGGTCGGGTT-3’		
β-actin	F 5’-CGTGCTGTGTTCCCATCTATC-3’	L08165	219 (150-368)
	R 5’-CTCCTCAGGGGCTACTCTCAG-3’		
σB gene for eukaryotic expression	F 5’-GCAAGCTTGCCACC ATGGAGGT ACGTGTGCCAAACTTTC -3’	KX421250	1104 (1-1104)
	R 5’-GCGGATCC TTACCAACCACAC TCCACAACAGTG -3’		
σB gene for qRT-PCR	F-5’-GGAGGTACGTGTGCCAAACT-3’	KX421250	150 (3-152)
	R-5’-CAACAATACGCATTGCCAAC-3’		

qRT-PCR using β-actin as internal control experiment was used for validation of arthritic mechanism of ARV σB protein as revealed by microarray.

### Gene expression profiling in ARV infected and σB transfected cells

The dysregulated genes were identified taking Paired t-test for statistical analysis (fold change ≥ 2 and p value <0.05). A total of 3841 genes were upregulated and 2868 downregulated in ARV infected cells, whereas 2194 were upregulated and 1832 downregulated in σB transfected cells.

### Prediction of canonical networks and DEGs activation

**Figure 3:**
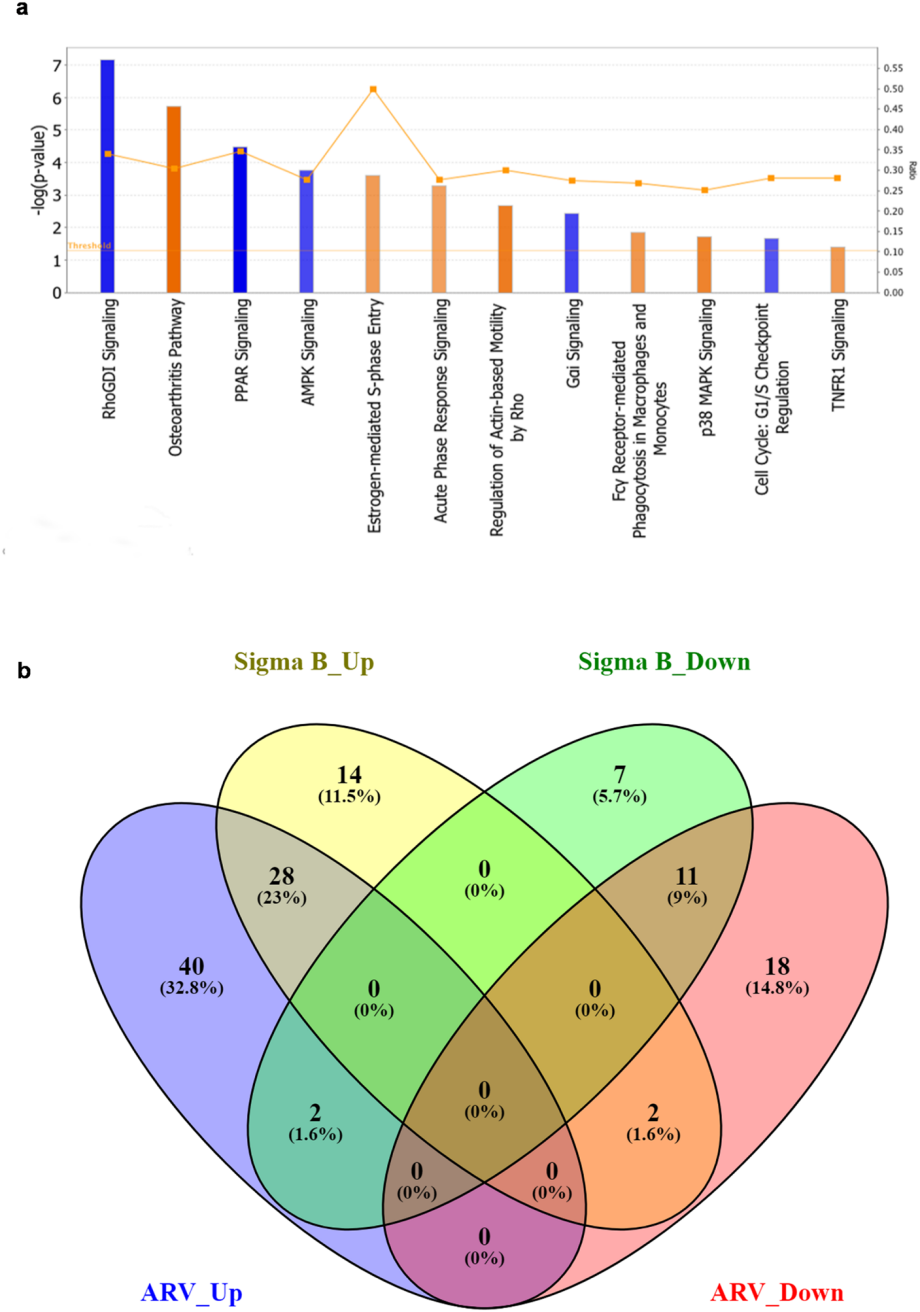
Activated canonical pathways in ARV infected and pDsRed-Express-N1-σB transfected groups **(a)** Depicts the activated canonical pathways based on their z-score ≥2 analysed through IPA, **(b)** Venn diagram of differentially expressed genes (DEGs). All DEGs are clustered into four comparison groups represented by four ellipses. The sum of all the figures in one ellipse represents the number of DEGs in one comparison group (e.g., ARV infected vs. σB transfected). The overlapping parts of different ellipses represent the number of DEGs in common from those comparison groups. Diagram shows the upregulated and downregulated unique and common genes related to osteoarthritis pathway of both ARV infected and σB transfected CEF cells.

**Figure 4:**
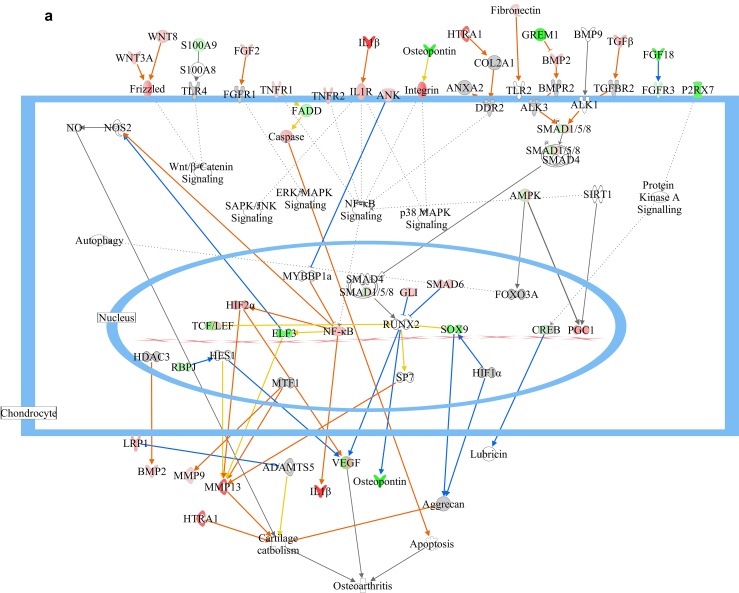

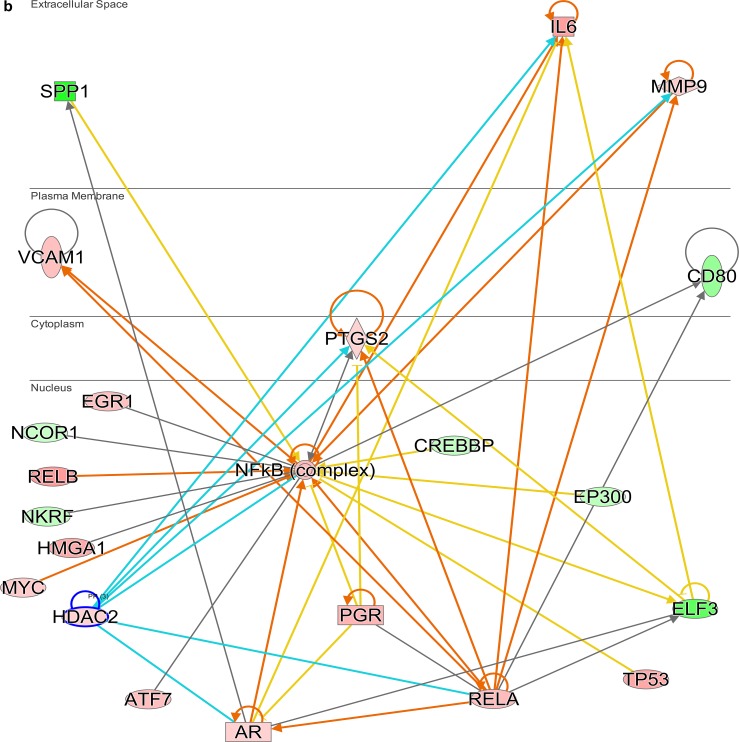
Delineated mechanism of osteoarthritis changes in response to σB protein **(a)** Pathway contains the differentially expressed genes related to ARV infected cells showing their mode of action and relation with each other in arthritis activation. **(b)** Predicted mechanism of arthritis induced by σB protein by NFkB pathway and the up and down regulation of closely related genes of NFkB network. Red colour indicates the upregulation of particular gene and green colour indicates the downregulation of particular gene. Blue line indicates leads to inhibition and orange line indicates lead to activation and orange line indicates “finding inconsistent with state of downstream molecule”.

The IPA analysis was done by taking –log (p-value) threshold at 1.3 and z-score at 2. Taking apoptosis, cellular immune response, humoral immune response and pathogenesis into consideration activated canonical pathways of σB transfected cells were predicted and most of them were related to pathogenesis and apoptosis revealing the catastrophic effect of σB in host (Figure 3a). Venn diagram of differentially expressed genes (DEGs) is shown in (Figure 3b). The most important prediction that was shown was the activation of osteoarthritis pathway with z-score 3.151, which was correlating with the previous research finding i.e. association of ARV in teno-synovitis syndrome (Figure 4a). The other enriched pathways like “Role of IL-17A in Arthritis” with –log (p-value) 1.64 also validated the association of σB in arthritis.

### Analysis of expression of upstream regulators of osteoarthritis pathway

IPA analysis showed the inhibition and activation of molecules that causes arthritis in poultry. Casp 1 and Casp14 were upregulated in both virus and σB treated group which showed the activation of apoptosis that may lead to activation of osteoarthritis. Likewise upregulation of ANK2, in both the groups was predicted to inhibit MYBBP1, which was leading to development of osteoarthritis by NF-κB activation through activation of IL-1β similar to activation of TLR2 by upregulated fibronectin. Additionally, activation of MMP13 by SP7 led to activation of cartilage catabolism with HTRA1. Moreover SOX9 predicted to inhibit aggrecan directly or indirectly through inhibition of HIF1α that activates cartilage catabolism was found to be downregulated. NF-κB complex played a vital role in progression of osteoarthritis through RELA and VCAM1 which was linked with osteoarthritis as a precursor [34]. The predicted pathway (Figure 4b) also correlated the Histone Deacetylase 2 (HDCA2) with IL6 and MMP9 and RELA which was seem to be boost the progression of osteoarthritis by repressing cartilage specific gene [35]. The previous experiment suggested the involvement of IL6 in osteoarthritis progression through MMP13 [36], which is further established in current study showing IL6 as upregulated DEG in both virus and σB treated group.

### Comparison analysis

Among total 122 DEGs of osteoarthritis pathway, 28 upregulated and 11 downregulated DEGs were common to both virus treated and σB transfected cells. Moreover 14 upregulated and 7 downregulated were unique in σB transfected cells. The common DEGs showed association of σB in osteoarthritis in relation to ARV. However, two genes in σB transfected cells have reverse pattern of regulation (Up to down & down to up regulation) than ARV infected cells (Figure 3b).

### Protein-protein interaction network among the differentially expressed genes

**Figure 5:**
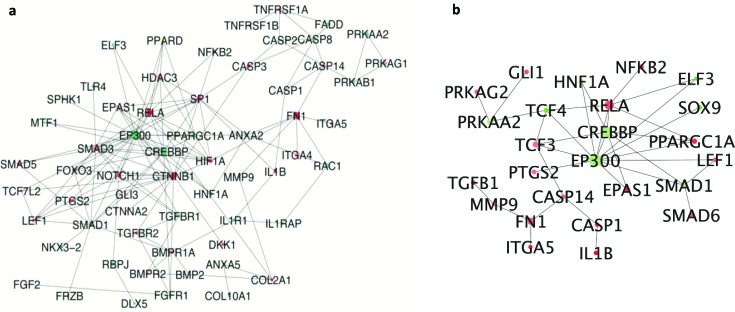
Interconnected network of differentially expressed osteoarthritis related genes **(a)** DEGs of ARV infected CEF cells compared to whole DEGs. Each circle indicating the node or member genes of the network are related to the hub i.e. with higher diameter than other arranged according to their degree; **(b)** DEGs of σB transfected CEF cells compared to whole DEGs. Each circle indicating the node or member genes of the network are related to the hub i.e. with higher diameter than other arranged according to their degree.

Predicted DEHC networks of proteins involved in activated osteoarthritis pathway in virus infected and σB transfected cells are shown. The network analysis in σB transfected cells showed the interaction between the major proteins viz. EP300, CREBBP, SMAD1, SOX9 that were downregulated and TGFB1, NFKB2, RELA, SMAD6, CASP1, IL1B that were upregulated (Figure 5b). EP300 and CREBBP were found to be major hubs with degree 384 and 254, respectively. RELA was highly connected upregulated gene with degree 231 and found to be connected with NFKB2, CREBBP and EP300. CREBBP, important for chondrogenesis was highly connected with SMAD1, which was mostly associated with chondrocytes differentiation. In virus, EP300 and CREBBP was also downregulated and highly connected with degree 447 and 292 respectively. RELA was upregulated and connected with degree 265 (Figure 5a).

### Validation of microarray data by qRT-PCR

**Figure 6:**
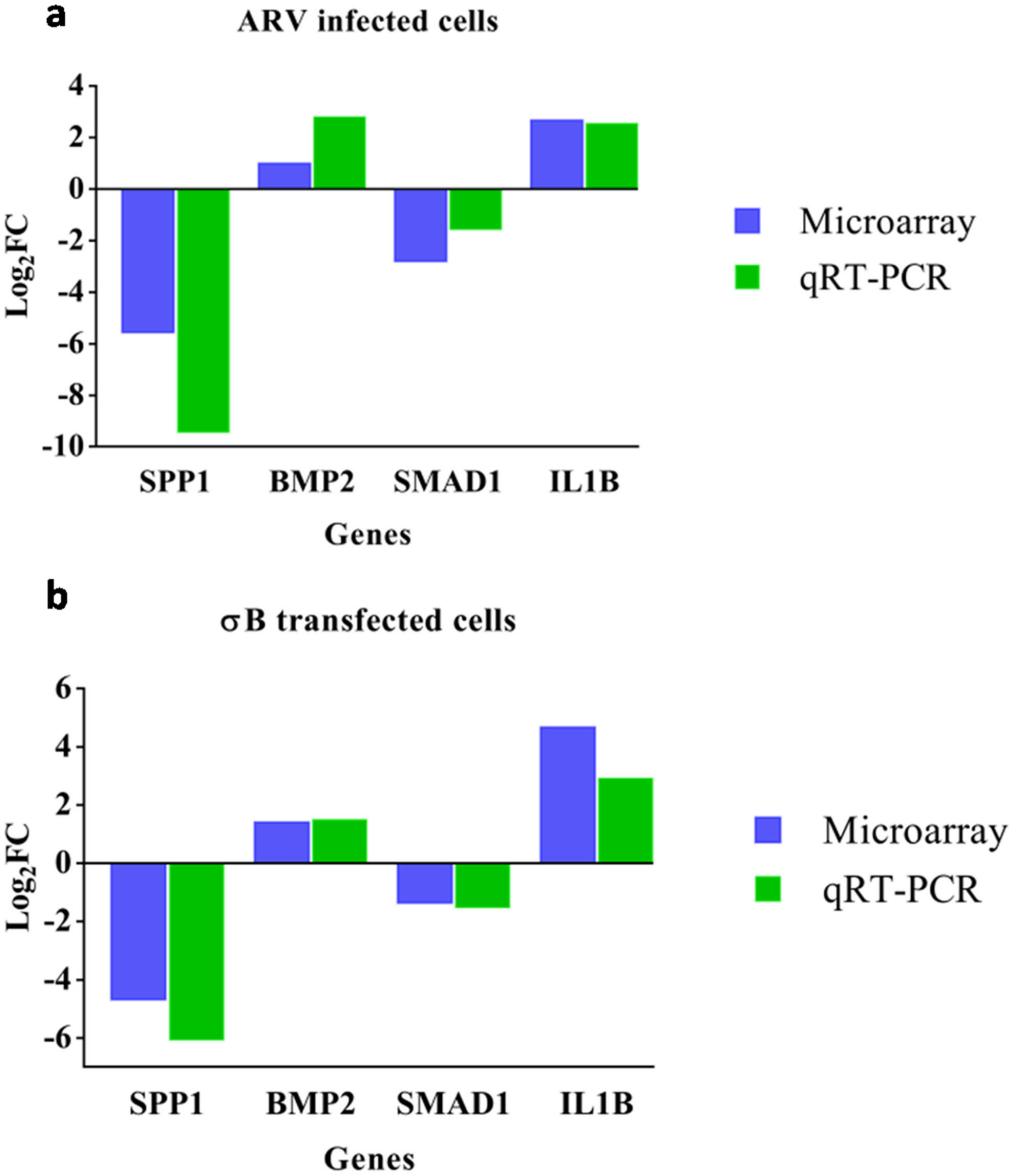
The Y-axis of the bar graph indicates the log2 fold changes of respective gene in ARV infected **(a)** and σB transfected **(b)** CEF cells. cDNA derived from CEF cells at 48hr of infection/transfection was compared to respective control by qRT-PCR and microarray.

Microarray analysis data of ARV infected cells (Figure 6a) and σB transfected cells (Figure 6b) were validated by using primers of 4 important candidate genes (SPP1, BMP2, SMAD1 and IL1B) that were involved in osteoarthritis pathway. β-actin was used as endogenous control. The results of the qRT-PCR validation were in concurrence with the microarray results.

## DISCUSSION

ARVs belongs to *orthoreovirus* genus of Reoviridae family. It has been found to be associated with tenosynovitis, runting-stunting syndrome of chickens [37]. It is a non-enveloped virus with icosahedral symmetry containing 10 genome segments. According to their electrophoretic mobility the genomic segments can be separated into large (L-class), medium (M-class), small segments (S-class). Most importantly s-class genome plays important role in inflammation, apoptosis and serodiagnosis. σB protein is found to be more conserved than other S-class proteins among ARV genes [38], but yet there is no report signifying the of role of σB in pathogenicity of ARV infection. This present *in vitro* study was carried out to examine pathogenesis related gene expression of host, in response to ARV infection and σB transfection. σB protein was specifically chosen and to elucidate its effect towards provoking pathogenesis in host vis-à-vis host response to virus. Insight into the signaling pathways and key signaling molecules involved in tissue level defense mechanisms against ARV infection can help to elucidate the response of the host to ARV infection.

Previously, it was reported that ARV and σC protein induces DNA damage and this process involves ROS intermediates and signaling pathways associated with DDIT-3 and GADD45α using microarray [31]. Another high throughput next generation sequencing study revealed whole genome sequence of two ARV isolates and established that the two co-infection strains showed close relationships in σA, σB and σNS genes [32]. Most recently a report identified that Wnt14 plays a pivotal role in initiating synovial joint formation in the chick limb, but the researchers were unable to determine the specific pathway that is responsible for the joint pathogenesis of ARV [39]. It was shown that the elevated expression of Wnt14 gene was observed, which might play a key role in the development of the disease [33]. The detailed study regarding the molecular arthritic pathogenesis and role of σB protein in viral arthritis was still unknown. In our study, the Ingenuity® Pathway Analysis (IPA®) was used for detailed study of pathways involved in osteoporosis. The detailed study was performed showing various pathways involved in up and down regulation of DEGs in ARV virus infected and σB transfected chicken embryo fibroblast cells.

Despite of many studies towards diagnosis and pathogenesis induced by ARV, there was no study signifies the pathway of in ARV and σB protein induced joint pathogenesis, which was the main objective of our study. One colour microarray based gene expression analysis experiment was performed and the differentially expressed genes in ARV infected and σB plasmid transfected CEF cells were identified by analysing the feature extraction data using GeneSpring V14.5. Based on the (FC) ≥2 and p value <0.05, the differentially expressed genes in both the groups were identified. In ARV infected vs control group 3841 DEGs were upregulated and 2868 DEGs were downregulated, where as in case of σB transfected vs control group 2194 and 1832 DEGs were upregulated and downregulated respectively.

Osteoarthritis is the progressive degradation of joints such as knee, with an endpoint of pain and complete loss of function. During osteoarthritis also involves other tissues of the joint, such as synovial fibroblasts and immune cells. During osteoarthritis chondrocytes undergo complex changes, including extensive degradative enzyme secretion, hypertrophy, and apoptosis, along with the formation of osteophytes from other mesenchymal stem cells [40]. This closely resembles the normal developmental transition from cartilage to bone, which has thus served as a major focus and model of research into the molecular mechanisms of osteoarthritis. The pro-osteoarthritis pathways generate autocrine self-activating loops in response to arthritic disease like ARV infection which can, unless countered by other events, can degrade cartilage to the point of symptomatic decline and destruction.

The major known molecular axes of osteoarthritis are the protective TGFβ to SOX9 pathway, which maintains cartilage, and the IL-1β and other inflammatory signals to RUNX2 pathway, which respond to inflammatory and damage signals to prompt repair, expression of degradative enzymes, and development towards bone including hypertrophy and apoptosis of chondrocytes. IL-1β was highly upregulated in σB transfected cell with 4.57 log2 (FC) showing increased inflammatory response, which suggested its catabolic effect on cartilage extracellular matrix along with TNFα [41]. Proinflammatory cytokines regulate the expression of a number of proteinases which destroy the extracellular cartilage [42]. Metalloproteinase and aggrecanases tend to degrade cartilage [43]. MMP-13, a matrix metalloproteinase expressed in osteoarthritis cartilage and causes collagen degradation, was upregulated in σB treated group [44, 45] MMP2, was upregulated in both ARV and σB treated group, is known as the most potent type II collagen degrading protein and along with MMP-13. MMP-2 was also significantly detectable in late stage osteoarthritis [46]. Previous study reported the association of IL1RN with IL-1β in osteoarthritis, suggesting that the upregulation of IL1RN in σB transfected cell, can increase the occurrence of osteoarthritis [47]. Moreover, IL-6 which was upregulated in both σB transfected and virus infected cells, seems to be associated with osteoclastogenesis [48, 49] indicating its role towards arthritis stimulation.

Like IL-1β, TGF-β is a regulator of both anabolic and catabolic activity in joint. With combined effect, TGF-β plays catabolic activity along with ESR1 and its association was also seen in cartilage loss, enhanced matrix synthesis or adaptive responses of bone to degenerate cartilage [50]. The TGF-β signalling induces arthritis through activation of ALK1, which acts through Smad 1/5/8 pathway [51]. Also, estrogen association was studied with IL-1β for modulating the expression of MMPs [52]. The previous study also showed the association of MMP-13 expression with ALK1 expression suggesting them as key molecules of chondrocyte hypertrophy and osteoarthritis [53]. Similarly in our study, IL-1β, TGF-β were upregulated whereas Smad 1 is downregulated in both virus and σB treated group leading to altered metabolism in joints leading to arthritic changes.

BMP which was upregulated in both virus and σB treated groups is associated with condensation and chondrocyte differentiation through SOX9 expression through Smad pathway [54, 55]. Further, our data in σB treated group showed downregulation in expression of SOX9 gene, a protective and stabilizing factor for chondrocytes, which glimpse towards activation of arthritis, which was also proofed by downregulation of CEBP/p300 in virus infected group which was associated with chondrogenesis [56]. BMP signalling was studied to be inhibited by Smad6 [57] its upregulation in σB treated group suggested its role in progression of arthritis. The upregulation of Smurf1 seems to boost the activity Smad6 and showed delay in endochondral ossification with Smad6 [58]. The experimental data suggested upregulation of Smurf1 both in ARV and σB treated groups. Association of Smurf1 with Smad6 inhibits Smad 1/5/8 signalling pathway (associated with BMP pathway) resulted in blocking BMP-2 induced chondrocyte hypertrophy and chondrocyte terminal differentiation terminating bone formation [59]. This hypothesis was also enlightened further with upregulation of COL9A2, which shows increase in its expression in arthritis [60] and TLR2, which will upregulate MMP increasing collagenolysis and aggrecanolysis [61, 62]. TLR2 was found to be upregulated in both virus and σB treated group. Collectively, our study suggests the probable mechanism of joint pathogenesis in ARV infection and the role of σB gene in regulation of the arthritis pathway.

From this comprehensive study, we can conclude the molecular pattern of expression and activation of osteoarthritis pathway genes by ARV and its σB protein. The current study, strengthen our understanding of the ARV induced joint pathogenesis and establishes the role of σB protein in runting/stunting syndrome which in-turn may help in devising suitable treatment/control strategy for ARV infection.

## MATERIALS AND METHODS

### Preparation of CEFs and virus propagation

CEFs were freshly prepared from 10-day-old chicken embryos [63, 64] and were grown in DMEM (Gibbco) with 10% fetal calf serum (FCS) for ∼ 24 hours at 37°C under 5% CO_2_ and then maintained at 5% FCS. The CEFs in 4 well plates were infected at a multiplicity of infection (MOI) 15 of ARV (DVB02) for 48 h at 37°C and 5% CO_2_ and mock infected CEF cells in DMEM acted as control.

### Preparation of eukaryotic construct

#### Amplification of σB gene

The σB gene for eukaryotic expression was amplified from Champion pET SUMO-σB as template using specified primer set [25] with slight modification by introducing kozak sequence and *Hind*III and *Bam*HI restriction enzyme site (Table 1). The PCR products were analyzed by 1.2% agarose gel electrophoresis after staining with Ethidium bromide (0.5μg/ml) under Gel Doc System.

### Restriction enzyme digestion and cloning of σB gene in pDsRed-Express-N1

The PCR product was purified by QIAquick^®^ Gel Extraction Kit (Qiagen, Germany) as per manufacture's protocol. The gel purified σB gene and pDsRed-Express-N1 vector was digested with *Hind*III and *Bam*HI (NEB, USA) and ligated by T4 DNA ligase (NEB, USA) with standard protocol in 1:3 ratio of vector to σB.

### Screening of σB positive recombinants

The ligated product was transferred into *E. coli* DH5α strain by calcium chloride and heat shock transformation using a standard protocol. The transformed cells were plated on LB agar plates containing Kanamycin (50 μg/ml) and incubated overnight at 37°C. The plates were observed for presence of colonies. For screening of positive recombinants the colony PCR was done with specified primer sets and protocols [25]. The PCR products were seen in 1.2% agarose electrophoresis with ethidium bromide (0.5μg/ml) under Gel Doc System. The positive clone was again confirmed by restriction enzyme digestion with *Hind*III and *Bam*HI.

### Transfection of recombinant plasmid in CEF cell

pDsRed-Express-N1-σB clones were transfected in 70-80% confluent cells in a four well plate using Lipofectamine 2000 (Invitrogen, USA) as per manufacturer's protocol with slight modification and kept for 48 h at 37°C and 5% CO_2_. Western blot analysis was done to show the expression of σB protein by taking out the cells from tissue culture plates using mammalian protein extraction reagent (Genetix, India) according to manufacturer's protocol. The western blot was performed with standard protocol with slight modification of blocking the membrane overnight with 5% skim milk in TBST at 4°C. The optimum dilution of chicken raised hyperimmune sera 1:200 and the HRP conjugated anti-chicken secondary antibody at 1:2000 for western blot was determined by checkerboard analysis.

### Experimental groups

For microarray experiment the CEF cells were grown in 4 well plates. For virus treatment group cell culture wells in triplicate were infected at MOI 15 of ARV strain DVB02. Mock infected CEF cells in DMEM acted as control for virus treatment group.

For σB treatment group cell culture wells in triplicate were transfected with 2.0μg of pDsRed-Express-N1-σB construct using Lipofectamine 2000. Empty pDsRed-Express-N1plasmid transfected CEF cells were used as control for σB transfection group. CEF cells for all the groups were kept for 48 h at 37°C in 5% CO_2_ concentration. After 48 h of incubation the cells from three wells of each treatment and control group were harvested and pooled separately and subjected RNA isolation and RNA quality estimation using bioanalyzer. The experiment was repeated after a gap of few days and RNA collected from two independent experiments was subjected to microarray analysis.

### RNA isolation and array hybridisation

RNAs were extracted using the RNeasy mini Kit (Qiagen, Germany) according to the manufacturer's protocol from 4 different groups as virus infected, control cells, pDsRed-Express-N1-σB transfected cells and pDsRed-Express-N1 transfected cells. Sample RNAs were quantified using a NanoDrop ND-1000 and maintained at -80°C for further use. For the microarray analysis, RNA quality was assessed using an Agilent Bioanalyzer (Agilent Technologies, USA). Sample RNA integrity numbers (RINs) were obtained to assign values to RNA measurements in an unambiguous manner. Total 200ng RNAs were reverse transcribed to produce double-stranded cDNA, from which cRNAs were synthesized and then labeled with cyanine-3-CTP. The labeled cRNAs were hybridized onto Agilent Chicken Gene Expression (4^*^44K, Design ID: 026441) microarrays [65]. After washing, the arrays were scanned using an Agilent SureScanner G2600D (Agilent Technologies, US). The sample labeling, microarray hybridization and washing were performed based on standard protocols provided by the Agilent Technologies, USA.

### Microarray data analysis

To analyse array images, raw data were extracted using Feature Extraction software (version 11.5.1.1, Agilent Technologies) and then analyzed and normalized using the quantile algorithm. GeneSpring (version 14.5, Agilent Technologies, USA) was employed to perform a basic analysis of the raw data taking mock infected CEF cells as control for ARV infected cells and pDsRed-Express-N1 transfected cells as control for pDsRed-Express-N1-σB transfected cells to normalize intensity and to give fold change. The microarray data have been submitted to the GEO database (http://www.ncbi.nlm.nih.gov/geo/) under accession number GSE103067. Differentially expressed genes were then identified based on fold-changes and P-values calculated using paired t-test. The threshold for up- and down-regulated genes was a fold change > = 2.0 and a P value < 0.05.

### IPA analysis

The DEGs of both groups were uploaded into QIAGEN's Ingenuity® Pathway Analysis (IPA, QIAGEN Redwood City, www.qiagen.com/ingenuity) and both core and comparison analysis was performed to identify canonical pathways, various upstream regulators such as transcription factors (TFs), mature microRNAs (miRNAs) and downstream effects pertaining to ARV infection by causal analysis approach which is based on the Ingenuity pathway knowledge base (IPKB) [66]. Fisher's exact test was used to calculate a p-value determining the significance of association between the differentially expressed genes and the canonical pathway. The significance was set at a p-value of 0.05. In a network, two genes are considered to be connected if there is a path (line called as edge) in the network between them. The gene or gene products are referred to as nodes and the intensity of the node color indicates the degree of up- (red) or down- (green) regulation of a given gene. Nodes are represented with various shapes to distinguish the functional class of the molecule. Labels on the edges describe the nature of the relationship between the nodes and genes. The n-value overlap p-value is computed based on significant overlap between genes in the dataset and known targets regulated by the transcriptional regulator.

### Protein-protein interaction network among the differentially expressed genes

BioGRID (Biological General Repository for Interaction Datasets) is an online interaction repository with data compiled through comprehensive curation efforts. It provides a comprehensive resource of protein–protein and genetic interactions for all major model organism species [67]. All interaction data are freely provided through search index and available via download in a wide variety of standardized formats. In this repository, protein-protein interactions in chicken are chosen. Customized Perl scripts were used to find out interactions involving the differentially expressed genes. DEHC – DEHC were identified by taking the fold change file and the specified pathway related genes that were identified using IPA and the interaction network was visualized in Cytoscape 3.0.2 [68].

### qRT-PCR validation of microarray results

After the RNA extraction according to manufacturer's instructions, 128 ng RNA was reverse transcribed to cDNA with RevertAid Reverse transcriptase Kit (Invitrogen) according to the manufacturer's instructions. The qPCR validation was performed with SYBR green (Maxima SYBR green/ROX qPCR Master Mix (2X)). The target genes and their respective primers are listed in Table 1. Reaction mixes were prepared according to the manufacturer's instructions, and each sample was run in triplicate in the 7500 Fast Real-Time qPCR machine (Applied Biosystems) with slight modification in annealing temperature at 58°C.

The data obtained from the qRT-PCR was analyzed to calculate the fold change. The fold change of individual mRNA was calculated based on the 2^-ΔΔCt^ method [69]. 2^-ΔΔCt^ value obtained was converted to natural logarithmic scale to make the data comparable with the microarray data. β-actin was used as a house keeping gene (endogenous control) [70, 71] for the analysis of data [ΔΔCT= (CT of infected/transfected cells - CT of β-actin in infected/transfected cells) - (CT of control cells - CT of β-actin in control cells)].

## Abbreviations

ARV: Avian Reovirus
CEF: Chicken Embryo Fibroblast
DEGs: Differentially expressed genes
DEHC: Differentially Expressed Highly Connected genes
DNA: Deoxyribonucleic acid
DMEM: Dulbecco’s Modified, Eagle’s Medium
dsRNA: double-stranded RNA
ELISA: Enzyme-linked immunosorbent assay
IL: Interleukin,
IPA: Ingenuity Pathway Analysis
LB Agar: Luria Bertani, Agar
RNA: Ribonucleic acid
SDS-PAGE: Sodium, dodecyl sulfate polyacrylamide gel electrophoresis
